# Anthropometric Markers With Specific Cut-Offs Can Predict Anemia Occurrence Among Malaysian Young Adults

**DOI:** 10.3389/fphys.2021.731416

**Published:** 2021-09-16

**Authors:** Sulagna Dutta, Ivan Rolland Karkada, Pallav Sengupta, Suresh V. Chinni

**Affiliations:** ^1^Department of Oral Biology and Biomedical Sciences, Faculty of Dentistry, MAHSA University, Jenjarum, Malaysia; ^2^Physiology Unit, Faculty of Medicine, Bioscience and Nursing, MAHSA University, Jenjarum, Malaysia; ^3^Department of Biotechnology, Faculty of Applied Sciences, AIMST University, Bedong, Malaysia

**Keywords:** anemia, anthropometry, body-mass index, hemoglobin, Malaysian adult

## Abstract

**Objective:** Anemia bears a high global prevalence with about 1.6 billion people living with this affliction. Malaysia carries the burden of 13.8% anemia prevalence which urges for extensive research directed to its prediction and amelioration. This is the first study that aims to (a) propose simple non-invasive predictive anthropometric markers and their specific cut-off values for early prediction of anemia among the young adults in Malaysia, (b) provide anemia prevalence based on both gender and ethnicity among young adults of Malaysia.

**Method:** The present cross-sectional study included 245 participants (113 men and 132 women) aged between 18 and 30 years. Anthropometric parameters were measured following the standard protocols. Blood samples were collected and hemoglobin levels were determined using the HemoCue haemoglobinometer (Hb 201+ System, Angelhom, Sweden) to detect the presence of anemia. The receiver operating characteristics (ROC) curve was employed to assess and compare the efficacy of anthropometric indices in the prediction of anemia. Data were analyzed using SPSS (v. 22.0, IBM, Chicago, IL, USA) and MedCalc (v. 19.05, Ostend, Belgium).

**Result:** The ROC analysis indicates that body mass index (BMI) is the best anthropometric marker with the highest area under the curve (AUC) and specificity (SP) for predicting the presence of anemia in young adults in Malaysia. Thus, the study proposes the optimal cut-off value of BMI for young men of Malaysia as 20.65 kg/m^2^ (AUC: 0.889) and young women of Malaysia as 19.7 kg/m^2^ (AUC: 0.904). The study also reports that Malaysian Indians have the highest prevalence of anemia (26.22%) followed by Malays (21.54%), “Others” (indigenous ethnic group) (20%), and Chinese (14.5%), with an overall higher prevalence of anemia in young adult women (21.96%) than in men (18.6%) of Malaysia.

**Conclusion:** The proposed anemia-predictive anthropometric markers with optimal cut-off values will aid early detection of anemia among young adults in Malaysia, and given its simple, inexpensive, and intelligible approach, it can be widely used. The ease of anemia prediction together with the reported distribution of anemia prevalence based on gender and ethnicity will facilitate in gauging the necessary extent of strategies of anemia management in the young adult population of Malaysia.

## Introduction

Anemia afflicts one-fourth of the global population (WHO, 2011), and Malaysia holds the burden of 13.8% anemia prevalence with 20.1% women and 4.9% men population being anemic (Abdullah et al., 2020). As per the WHO, anemia is a condition marked by hemoglobin level lower than the physiological value, by two SDs, in age- and gender-matched populations. WHO stipulated reference hemoglobin values for defining anemia are 13 and 12 g/dL for adult men and women, respectively (WHO, 2011).

Anemia is a multideterminant condition among which the vastly reported determinants are the demographic factors, such as age, gender, ethnicity (Yusof et al., 2018), geographical location (Bharati et al., 2015), physical activity (Choudhary and Binawara, 2012), and nutritional status (Aigner et al., 2015). A large cohort of research has exploited multitudinous causative factors of anemia and its prevalence in Malaysia (Ishak and Hassan, 1994; Hassan et al., 2005; Haniff et al., 2007; Khambalia et al., 2011; Nik Rosmawati et al., 2012; Soh et al., 2015). As anemia is a common blood disorder affecting the mass population, a multiethnic nation, such as Malaysia, must direct its research to understand the pattern of anemia prevalence based on ethnicity to develop effective management strategies. Studies on the middle-aged and older population of Malaysia have reported that anemia is most prevalent in Indians, followed by Malays, and Chinese (Yusof et al., 2018; Abdullah et al., 2020). However, the relationship between ethnicity and anemia among young adult population in Malaysia is yet to be determined. On the global aspect, reports pertaining to prevalence of anemia based on the ethnicity are scanty, but some of the studies revealed significant variations of occurrence of anemia among various ethnic groups (Frith-Terhune et al., 2000; Guralnik et al., 2004; Gaskell et al., 2008; Yusof et al., 2018). This can be explained by the differences in lifestyles, customs, and beliefs among the ethnicities which influence their dietary choices (Gaskell et al., 2008; Hiza et al., 2013; Nohan et al., 2020).

Early detection of anemia may lead to its rapid amelioration. Blood hemoglobin estimation is not a feasible option to predict the occurrence of anemia in the entire large population of a nation affected by it, and the complexity of prediction of anemia among the mass hinders to sketch-out proper rapid remedial strategies. In this aspect, it may be considered that anemia holds a close association with body type and various anthropometric parameters (Micozzi et al., 1989; Smith et al., 1991; Collett-Solberg et al., 2007; Saxena et al., 2011; Chang et al., 2014; Sinha and Haldar, 2015; Virginia and Fenty, 2017; El-Shafie et al., 2020). Thus, anthropometric parameters can serve as surrogate predictors of anemia, which are simple, non-invasive, and rapid yet accurate. This will aid early speedy detection of anemia among a large population as the methods are inexpensive and do not mandate the presence of medical professionals. The most studied anthropometric parameters that find an association with hemoglobin levels include body mass index (BMI), height, weight, waist circumference (WC), waist-to-hip ratio (WHR), and waist-to-height ratio (WHtR) (Lee et al., 2004; Al-Hashem, 2007; Vuong et al., 2014). Nevertheless, to establish anthropometric parameters as predictors of anemia, it is needed to evaluate their definite predictive values. Thus, this study finds novelty in its aim to propose predictive anthropometric markers along with their specific cut-off values for early prediction of anemia among young adults in Malaysia and to provide the prevalence of anemia among adult men and women of Malaysia based on ethnicity.

## Materials and Methods

### Ethical Considerations

The Institutional Research Ethics Committee of MAHSA University (RMC/EC05/2019) has approved the study proposal. The subjects were randomly selected from different faculties of the MAHSA University, Saujana Putra Campus, Jenjarom, Selangor, Malaysia within the duration from December 1, 2018, to June 30, 2019. All the respondents provided their written informed consent to participate in this study.

### Study Population

This population-based cross-sectional study included 245 young adult men and women (comprised of 113 men and 132 women) aged between 18 and 30 years. The sample size was calculated considering the prevalence of anemia in Malaysia as 13.8% (Abdullah et al., 2020). The initial sample size, obtained using the formula, *n*_1_ = (*z*^2^PQ)/*d*^2^ (where, *n*_1_ = initial sample size, *z* = statistic corresponding to the level of confidence, *P* = expected prevalence, *Q* = proportion of exception, *d* = precision corresponding to effect size), was 182.6 (~183). After including 20% of sampling error, using the formula, *n* = *n*_1_ + (*n*_1_ × 20/100) (where *n* = final sample size), we obtained the final sample size as 219.6 (~220), and thus, the sample size of 245 used in this study is sufficed to represent the young adult population of Malaysia.

Data collected were segregated based on the ethnic groups of Malaysia: Malays, Chinese, Indians, and a residual “Others” category including all indigenous populations of Malaysia. The exclusion criteria for this study were (a) citizens of other countries, (b) married individuals, (c) individuals with any kind of blood coagulation-related disorders, (d) individuals who had a medical history of depression, stroke, angina, hypertension, asthma, diabetes mellitus, hypercholesterolemia, tuberculosis, hepatitis chronic, bronchitis chronic, kidney diseases, and cancer, and (e) individuals with genetic disorders, such as Turner's syndrome, primary hypopituitarism. Exclusion criteria also consist of subjects aged under 18 years and pregnant women.

### Anthropometric and Biochemical Measurements

Height, body weight, WC, and buttock circumference (BC) were measured for every subject, and it was ascertained that the subjects would be in minimal garments and bare feet on a plane surface. Body weight was measured using portable electronic scales (measured to the nearest 0.1 kg) and prior to every measurement, the instrument was reset to “zero” to attain accuracy. Portable stadiometers were used to measure the height (measured to the nearest 1 cm), and the subjects were instructed to keep their feet together with their shoulder blades, hips, and heels in line with and touching the instrument stick, and head held straight on the horizontal plane. Measurement of WC and HC were done using inextensible anthropometric tape and the subjects stood erect with arms resting at their sides and feet placed together (Legro et al., 1999). The BMI (or Quetelet Index) was determined by the formula, “BMI = weight (kg) / (Height in m)^2^” (WHO, 2006). Other anthropometric indices, WHR and WHtR were also calculated. The hemoglobin concentration was measured by following standard biochemical examinations. The capillary blood sample was tested for hemoglobin level using a HemoCue haemoglobinometer (HemoCue Hb 201+ System, Angelhom, Sweden).

### Terms Definition

Anemia is diagnosed using the hemoglobin values and the levels given by the WHO, i.e., <12 g per dL in women and <13 g per dL in men (WHO, 2011). Several studies have employed these WHO stipulated diagnostic criteria for screening of anemia (Cesari et al., 2004; Semba et al., 2009; Yusof et al., 2018; Abdullah et al., 2020). The present study also employed the same WHO criteria to include men and women subjects in the anemic study group.

### Statistical Analysis

Statistical analysis was performed considering a confidence level of 95%. The acquired data were analyzed using statistical software, namely, SPSS (v. 22.0, IBM, Chicago, IL, USA) and MedCalc (v. 19.05, Ostend, Belgium). For the continuous variables, the median (upper limit-lower limit) was determined as the frequency distribution of the parameters rejected normality. Descriptive statistics and the Students' independent sample *t*-test were employed, and a statistically significant level was set up at *P* < 0.05.

Receiver operator characteristic (ROC) analysis was done using body weight, BMI, WC, BC, WHR, and WHtR as continuous variables and anemia as the categorical variable. The area under the curves (AUCs), sensitivities (SS), specificities (SP), Youden's indices, and cut-off values for all continuous variables were obtained and compared. For all evaluations, *P* < 0.05 was deemed statistically significant.

## Results

### Anthropometric Parameters as Predictors of Anemia

**Figure 1:**
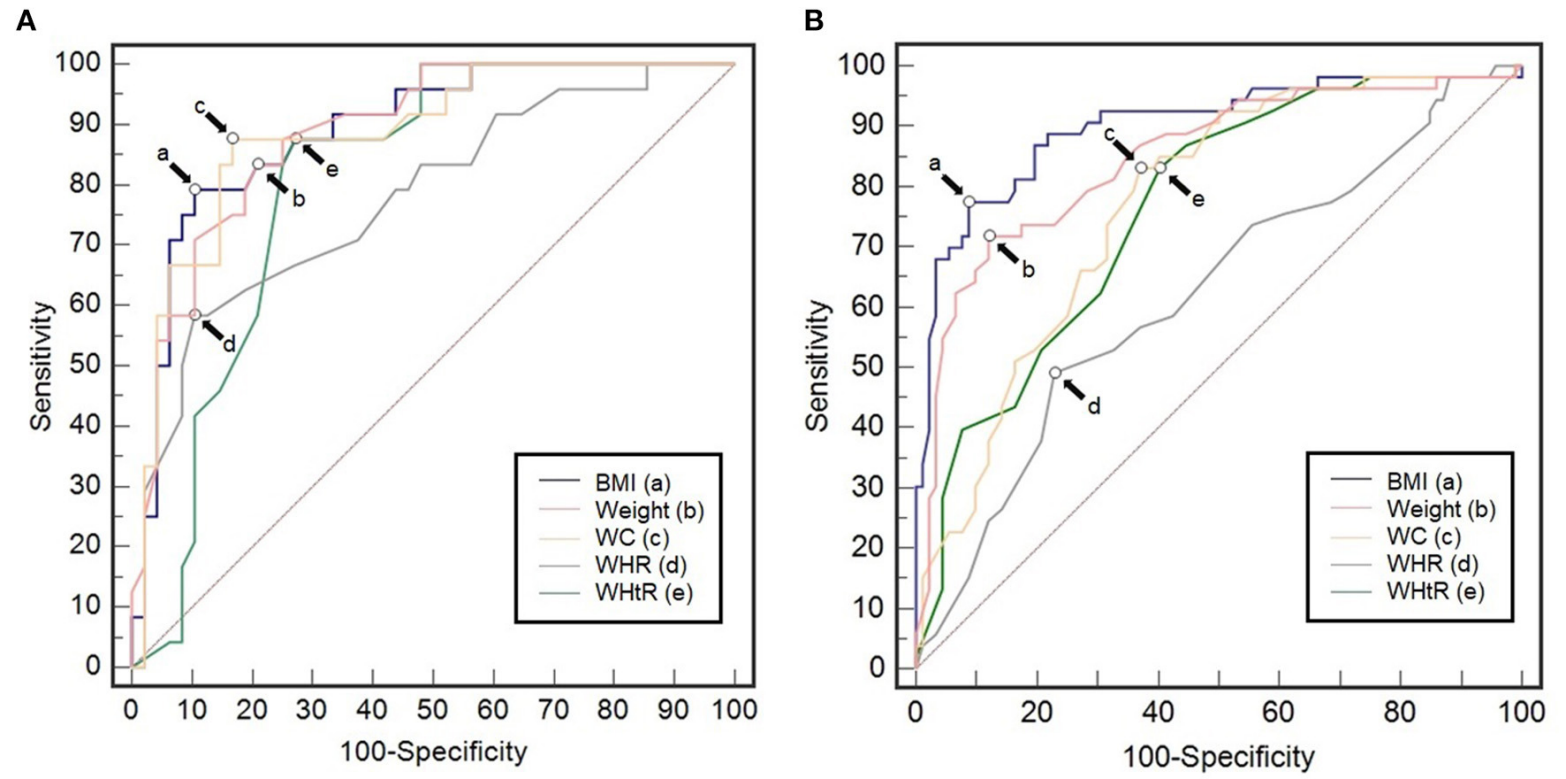
Receiver operating characteristics curves of body mass index, body weight, waist circumference (WC), waist-to to-hip ratio (WHR), and waist-to to-height ratio (WHtR) in the prediction of anemia among young adults of Malaysia **(A)** men (*n* = 113); *a* = 20.65, *b* = 62, *c* = 76, *d* = 0.79, *e* = 0.45, and **(B)** women (*n* = 132); *a* = 19.7, *b* = 49.5, *c* = 71, *d* = 0.74, *e* = 0.45.

The ROC curves of the anthropometric parameters, i.e., BMI, body weight, WC, BC, WHR, and WHtR for prediction of anemia in the young adults of Malaysia are presented in Figure 1. The ROC curve analysis revealed a high-predictive value of BMI for anemia, which in men had AUC (CI 95%) of 0.889 with the optimal cut-off value of 20.65 kg/m^2^ (sensitivity, SS: 79.17%; and specificity, SP: 89.80%), and in women, BMI had the AUC (CI 95%) of 0.904 with the optimal cut-off value of 19.7 kg/m^2^ (SS: 78.18%; SP: 91.40%). Following BMI, the predictive value of body weight for anemia in men, showed an AUC (CI 95%) of 0.886 with the optimal cut-off value of 62 kg (SS: 83.33%; SP: 79.59%), while in women, it showed AUC (CI 95%) of 0.836 with the optimal cut-off value of 49.5 kg with (SS: 69.09%; SP: 88.17%). WC had the cut-off values of 76 cm (AUC: 0.883; SS: 87.5%; SP: 83.33%) and 71 cm (AUC: 0.771; SS: 83.02% SP: 63.04%) for men and women, respectively. WHR showed the predictive cut-off values of 0.79 (AUC: 0.761; SS: 58.33%; SP: 87.76%) for men and similarly in women, it yielded cut-off value of 0.74 (AUC: 0.624; SS: 49.06%; SP: 77.17%). For WHtR, the predictive cut-off value for anemia inmen was 0.45 (AUC: 0.793; SS: 87.50%; SP: 71.43%), and in women, it was also 0.45 (AUC: 0.764; SS: 83.64%; SP: 59.14%).

Body mass index stands out as the most potent anthropometric index to be used in the prediction of anemia in men based on the highest AUC and SP obtained. However, pairwise comparison of all the anthropometric variables in predicting anemia showed statistically significant differences only for WC vs. WHR and WHtR. It indicates that the efficacy of the anthropometric variables considered in the study is not distinctly different in detecting anemia in men (Table 1). However, in women, pairwise comparison of these anthropometric variables reflected the significant difference in all the cases (except WC vs. WHtR) and indicated the higher efficacy of BMI in predicting anemia among young adult women of Malaysia (Table 2).

**Table 1:** Comparison of receiver operating characteristics (ROC) curves of anthropometric indices in predicting anemia in young adult men of Malaysia.

**Variables**	**Cut-off values**	**AUC(95% CI)**	***P* value**	**SS (%)**	**SP (%)**	**Pairwise comparison of ROC curves**		
						**Variables**	**AUC difference**	***P* value**
BMI^b^	20.65	0.889	<0.0001	79.17	89.80	BMI ~ WHtR	0.082	0.0709
						BMI ~ WHR	0.113	0.1144
BW^c^	62	0.886	<0.0001	83.33	79.59	BMI ~ BW	0.003	0.8571
						BMI ~ WC	0.008	0.8307
WC^d^	76	0.883	<0.0001	87.50	83.33	WHtR ~ WHR	0.031	0.6007
						WHtR ~ BW	0.079	0.0905
WHR	0.79	0.761	<0.0001	58.33	87.76	WHtR ~ WC	0.074	0.0363^a^
						WHR ~ BW	0.110	0.1099
WHtR	0.45	0.793	<0.0001	87.50	71.43	WHR ~ WC	0.105	0.0498^a^
						BW ~ WC	0.005	0.8829

b *kg/m^2^;*

c *kg;*

d *cm; BMI, body mass index; BW, body weight; WC, waist circumference; WHR, waist-to-hip ratio; WHtR, waist-to-height ratio; AUC, area under curve; SS, sensitivity; SP, specificity;*

a *P < 0.05*.

**Table 2:** Comparison of ROC curves of anthropometric indices in predicting anemia in young adult women of Malaysia.

**Variables**	**Cut-off values**	**AUC(95% CI)**	***P* value**	**SS (%)**	**SP (%)**	**Pairwise comparison of ROC curves**		
						**Variables**	**AUC difference**	***P* value**
BMI^b^	19.7	0.904	<0.0001	78.18	91.40	BMI ~ WHtR	0.137	0.0001^a^
						BMI ~ WHR	0.276	0.0001^a^
BW^c^	49.5	0.836	<0.0001	69.09	88.17	BMI ~ BW	0.053	0.0225^a^
						BMI ~ WC	0.129	0.0003^a^
WC^d^	71	0.771	<0.0001	83.02	63.04	WHtR ~ WHR	0.139	0.0001^a^
						WHtR ~ BW	0.084	0.0401^a^
WHR	0.74	0.624	0.0109	49.06	77.17	WHtR ~ WC	0.007	0.6138^a^
						WHR ~ BW	0.223	0.0001^a^
WHtR	0.45	0.764	<0.0001	83.64	59.14	WHR ~ WC	0.147	0.0001^a^
						BW ~ WC	0.076	0.0354^a^

b *kg/m^2^;*

c *kg;*

d *cm; BMI, body mass index; BW, body weight; WC, waist circumference; WHR, waist-to-hip ratio; WHtR, waist-to-height ratio; AUC, area under curve; SS, sensitivity; SP, specificity;*

a *P < 0.05*.

### Anthropometric Variables and Hemoglobin Concentration

#### In Malaysian Young Adult Men and Women

Comparison of anthropometric variables and hemoglobin concentration between age-matched young adult men and women of Malaysia are presented in Table 3. In this study, the numbers of total respondents were 113 for men and 132 for women, respectively. The group of men includes 30 Malays, 32 Chinese, 29 Indians, and 22 others. Similarly, the group of women includes 35 Malays, 37 Chinese, 32 Indians, and 28 others.

**Table 3:** Age, hemoglobin concentration, and anthropometric variables of respondents.

	**Men (*N* = 113)**	**Women (*N* = 132)**
Age^a^	21.0 (24.0–18.0)	19.0 (23.0–18.0)
Weight^b^	66.0 (97.0–49.0)	50.0 (80.0–40.0)
Height^c^	165.2 (175.0–161.0)	155.8 (171.0–148.0)
BMI^d^	22.4 (32.4–18.9)	20.9 (34.6–17.3)
Hemoglobin^e^	14.4 (16.4–10.5)	12.3 (14.5–7.9)
WC^c^	79.3 (108.7–64.0)	73.0 (97.0–59.0)
BC^c^	93.7 (117.4–82.2)	91.2 (115–75)
WHR	0.86 (0.93–0.77)	0.79 (0.89–0.69)
WHtR	0.49 (0.63–0.40)	0.47 (0.63–0.37)

*Data are represented as median (upper limit–lower limit);*

a *years*,

b *kg*,

c *cm*,

d *kg/m^2^*,

e *g/dl; BMI, body mass index; BW, body weight; WC, waist circumference; BC, buttock circumference; WHR, waist-to-hip ratio; WHtR, waist-to-height ratio*.

Table 4 represents a gender-wise comparison of these variables among different ethnic groups. One-way ANOVA was applied for the test at 95% CI, level of significance was considered when *P* < 0.05. Comparison of data of age-matched young men showed significantly lower hemoglobin in Indians and others. Anthropometric variables, such as WC and WHtR were also found to be significantly lower in other groups. The height of Chinese men was found to be slightly higher than other groups. In women, body weight, WC, and WHR were found to be lower in Chinese and others, whereas Indians have significantly higher BC and height than other ethnic groups.

**Table 4:** Comparison of various parameters between men and women of different ethnic groups.

	**Malay (** ***N*** **=** **65)**		**Chinese (** ***N*** **=** **69)**		**Indian (** ***N*** **=** **61)**		**Others (** ***N*** **=** **50)**	
	**Men (*N* = 30)**	**Women (*N* = 35)**	**Men (*N* = 32)**	**Women (*N* = 37)**	**Men (*N* = 29)**	**Women (*N* = 32)**	**Men (*N* = 22)**	**Women (*N* = 28)**
Age^b^	21.0 (24.0–18.0)	19.0 (23.0–18.0)	20.0 (25.0–16.0)	20.0 (23.0–17.0)	20.0 (23.0–17.0)	21.0 (26.0–17.0)	21.5 (24.0–20.0)	21.0 (24.0–18.0)
Weight^c^	66.0 (97.0–49.0)	50.0 (80.0–40.0)	66.0 (110.0–48.0)	53.0 (91.0–40.0)^a^	66.3 (92.0–41.0)	57.0 (118.0–34.0)^a^	64.0 (96.0–55.0)	52.0 (81.0–42.0)^a^
Height^d^	165.2 (175.0–160.0)	155.8 (171.0–148.0)	171.0 (185.0–160.0)^a^	160.0 (173.0–149.0)^a^	170.0 (187.0–157.0)	158.0 (168.0–150.0)	171.5 (179.0–162.0)	157.0 (168.0–145.0)
BMI^e^	22.4 (32.4–18.9)	20.9 (34.6–17.3)	21.9 (36.7–16.3)	19.9 (35.1–13.3)	21.7 (31.5–13.8)	21.7 (35.2–13.1)	21.7 (29.1–19.4)	19.9 (34.6–17.3)
Hemoglobin^f^	14.4 (16.4–10.5)	12.3 (14.5–7.9)	14.1 (17.2–7.3)	12.7 (14.8–7.6)	13.6 (16.9–9.2)^a^	12.5 (14.2–8.2)	13.5 (16.2–10.6)^a^	11.7 (14.1–10.0)
WC^d^	79.3 (108.7–64.0)	73.0 (97.0–59.0)	76.6 (101.0–63.0)	69.0 (96.0–59.0)^a^	76.8 (103.0–63.0)	74.8 (104.0–56.7)	75.9 (102.0–63.9)^a^	69.5 (92.5–57.0)^a^
BC^d^	93.7 (117.4–82.2)	91.2 (115.0–75.0)	92.7 (114.0–78.0)	90.1 (112.5–80.0)	93.7 (113.2–72.0)	95.0 (117.5–75.0)^a^	91.9 (113.0–79.9)	88.0 (109.5–82.0)
WHR	0.86 (0.93–0.77)	0.79 (0.89–0.69)	0.83 (0.99–0.74)	0.76 (0.92–0.67)^a^	0.87 (1.01–0.75)	0.80 (0.97–0.67)	0.83 (0.98–0.75)	0.74 (0.88–0.68)^a^
WHtR	0.49 (0.63–0.40)	0.47 (0.63–0.37)	0.45 (0.63–0.37)	0.43 (0.60–0.37)^a^	0.45 (0.61–0.36)	0.45 (0.69–0.36)	0.43 (0.63–0.34)^a^	0.45 (0.61–0.37)

*Data are represented as median (upper limit–lower limit);*

b *years*,

c *kg*,

d *cm*,

e *kg/m^2^*,

f *g/dl;*

a *compared to same gender, different ethnic group; P < 0.05; BMI, body mass index; BW, body weight; WC, waist circumference; BC, buttock circumference; WHR, waist-to-hip ratio; WHtR, waist-to-height ratio*.

#### Comparison Between Anemic and Non-anemic Groups of Young Adults of Malaysia

**Figure 2:**
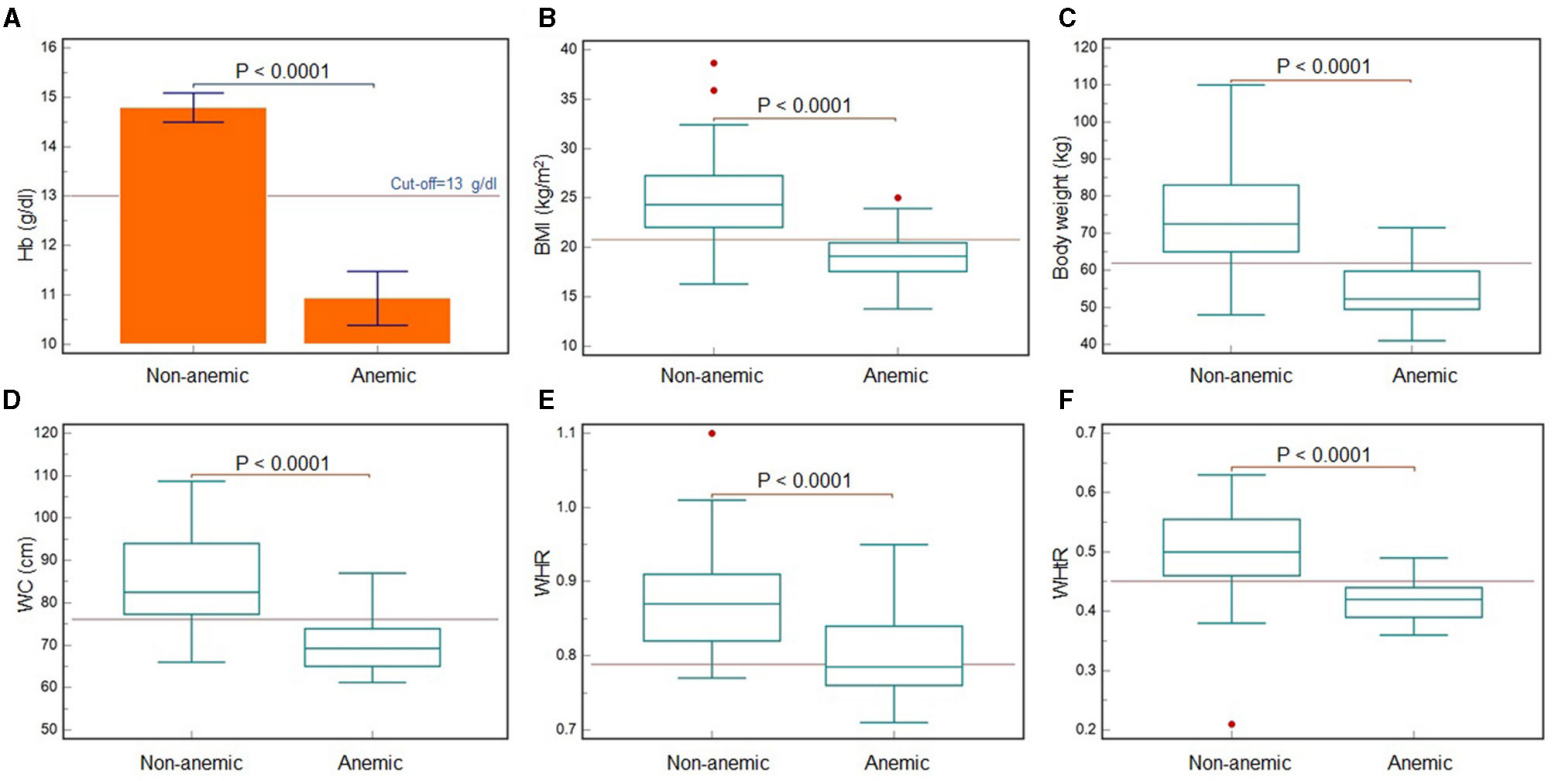
Comparison of hemoglogin levels **(A)** and anthropometric variables **(B–F)** between Malaysian young adult men with and without anemia. Statistically significant differences (*P* < 0.0001) were recorded for hemoglobin concentration **(A)**, BMI **(B)**, body weight **(C)**, WC **(D)**, WHR **(E)** and WHtR **(F)**. The red lines are indicating the cut-off values. Hemoglobin cut-off is as per the WHO reference standard (WHO, 2011).

**Figure 3:**
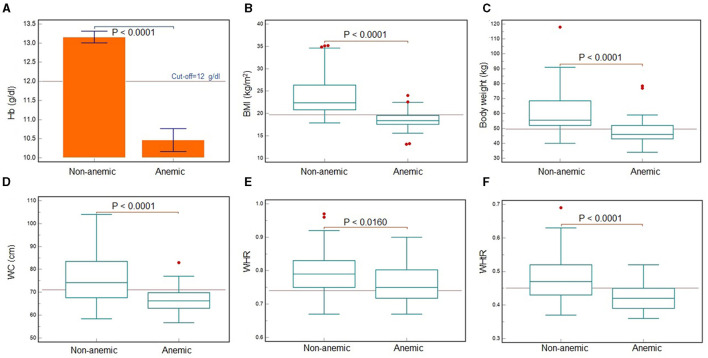
Comparison of hemoglogin levels **(A)** and anthropometric variables **(B–F)** between Malaysian young adult women with and without anemia. Statistically significant differences (all variables: *P* < 0.0001; WHR: *P* < 0.016) were recorded for hemoglobin concentration **(A)**, BMI **(B)**, body weight **(C)**, WC **(D)**, WHR **(E)** and WHtR **(F)**. The red lines are indicating the cut-off values. Hemoglobin cut-off is as per the WHO reference standard (WHO, 2011).

Among 113 men and 132 women, a total of 21 (18.6%) men and 29 women (21.96%) were found to be anemic following the WHO categorization (< 13 g per dL in men, < 12 g per dL in women) (WHO, 2011). Figure 2 shows significantly lower hemoglobin and all the other anthropometric variables in anemic men compared with normal (*P* < 0.0001). All the anthropometric variables (except height) were found to be significantly different between anemic and normal men, for all the ethnic groups. While the variables were compared across the ethnic groups, body weight was found to be significantly lower in Chinese men and others, whereas BMI was found to be significantly lower in Indian men (Table 5). For women too, all the anthropometric variables differed significantly between anemic and normal women (*P* < 0.0001) (Figure 3). Across the ethnic groups, Chinese women showed significantly higher body weight, but lower WHR (*P* < 0.05) (Table 6).

**Table 5:** Comparison of various parameters among the men of different ethnic groups.

	**Malay (** ***N*** **=** **30)**		**Chinese (** ***N*** **=** **32)**		**Indian (** ***N*** **=** **29)**		**Others (** ***N*** **=** **22)**	
	**Normal (*N* = 24)**	**Anemic (*N* = 6)**	**Normal (*N* = 28)**	**Anemic (*N* = 4)**	**Normal (*N* = 22)**	**Anemic (*N* = 7)**	**Normal (*N* = 18)**	**Anemic (*N* = 4)**
Age^c^	21.0 (24.0–18.0)	19.3 (20.0–18.0)	20.0 (23.0–17.0)	20.0 (25.0–16.0)	20.0 (23.0–17.0)	22.0 (22.0–20.0)	21.0 (24.0–18.0)	21.3 (23.0–20.0)
Weight^d^	66.0 (97.0–55.0)	51.1 (53.0–49.0)^a^	76.5 (110.0–48.0)	56.0 (71.5–48.0)^a^^b^	69.0 (92.0–53.0)	51.0 (56.5–41.0)^a^	66.0 (96.0–56.0)	54.0 (66.0–55.0)^a^^b^
Height^e^	167.0 (175.0–161.0)	164.5 (166.0–160.0)	172.0 (185.0–162.5)	168.0 (181.0–160.0)	169.5 (187.0–161.0)	170.0 (177.0–157.0)	170.5 (179.0–166.0)	169.5 (170.0–162.0)
BMI^f^	24.9 (32.4–20.2)	19.2 (20.7–18.9)^a^	24.4 (36.7–16.3)	19.9 (25.0–17.4)^a^	24.1 (31.5–19.1)	16.9 (19.6–13.8)^a^^b^	24.7 (29.1–21.2)	18.9 (22.0–19.4)^a^
Hemoglobin^g^	14.9 (16.4–13.2)	11.2 (11.9–10.5)^a^	14.7 (17.2–13.2)	11.6 (12.3–7.3)^a^	15.0 (16.9–13.2)	10.1 (12.1–9.2)^a^	14.5 (16.2–14.0)	10.8 (12.3–10.6)^a^
WC^e^	81.3 (108.7–72.0)	68.5 (70.7–64.0)^a^	82.5 (101.0–66.0)	69.9 (87.0–63.0)^a^	92.0 (103.0–66.5)	69.5 (74.0–63.0)^a^	84.0 (102.0–67.0)	68.9 (75.0–63.9)^a^
BC^e^	94.7 (117.4–89.0)	83.6 (87.3–82.2)^a^	100.2 (114.0–80.0)	87.8 (104.0–78.0)^a^	98.0 (113.2–89.0)	85.8 (87.0–72.0)^a^	96.9 (113.0–88.0)	86.9 (88.0–79.9)^a^
WHR	0.87 (0.93–0.81)	0.79 (0.80–0.77)^a^	0.85 (0.99–0.77)	0.78 (0.90–0.74)^a^	0.89 (1.01–0.81)	0.84 (0.95–0.75)^b^	0.88 (0.98–0.81)	0.79 (0.85–0.75)^a^
WHtR	0.50 (0.63–0.44)	0.42 (0.43–0.40)^a^	0.48 (0.63–0.38)	0.43 (0.49–0.37)^a^	0.51 (0.61–0.43)	0.42 (0.44–0.36)^a^	0.50 (0.63–0.45)	0.42 (0.44–0.34)^a^

*Data are represented as median (upper limit–lower limit);*

c *years*,

d *kg*,

e *cm*,

f *kg/m^2^*,

g *g/dl;*

a *normal vs. anemic*,

b *compared among normal/anemic across ethnic groups; P < 0.05; BMI, body mass index; BW, body weight; WC, waist circumference; BC, buttock circumference; WHR, waist-to-hip ratio; WHtR, waist-to-height ratio*.

**Table 6:** Comparison of various parameters among the women of different ethnic groups.

	**Malay (** ***N*** **=** **35)**		**Chinese (** ***N*** **=** **37)**		**Indian (** ***N*** **=** **32)**		**Others (** ***N*** **=** **28)**	
	**Normal (N = 27)**	**Anemic (*N* = 8)**	**Normal (*N* = 31)**	**Anemic (*N* = 6)**	**Normal (*N* = 23)**	**Anemic (*N* = 9)**	**Normal (*N* = 22)**	**Anemic (*N* = 6)**
Age^c^	19.0 (23.0–18.0)	20.0 (22.0–18.0)	20.0 (23.0–17.0)	21.0 (23.0–17.0)	20.0 (26.0–18.0)	21.0 (23.0–17.0)	21.0 (24.0–21.0)	20.0 (23.0–18.0)
Weight^d^	52.5 (80.0–44.0)	44.5 (53.0–40.0)^a^	54.5 (91–40.0)	49.3 (78.5–40.0)^a^^b^	68.0 (118.0–46.0)	44.5 (56.0–34.0)^a^	76.0 (81.0–49.0)	44.5 (77.0–42.0)^a^
Height^e^	155.3 (171.0–148.0)	156.5 (160.0–150.0)	159.5 (173.0–149.0)	160.3 (181.0–151.0)	158.0 (168.0–150.0)	157.0 (167.0–150.0)	159.0 (165.0–153.0)	156.0 (168.0–145.0)
BMI^f^	22.3 (34.6–17.9)	18.1 (20.7–17.3)^a^	21.8 (35.1–18.0)	18.7 (24.0–13.3)^a^	26.1 (35.2–19.9)	18.2 (20.1–13.1)^a^	27.9 (34.6–19.4)	19.2 (22.6–17.3)^a^
Hemoglobin^g^	12.8 (14.5–12.1)	9.4 (11.8–7.9)^a^^b^	13.1 (14.8–12.5)	10.9 (11.5–7.6)^a^	13.1 (14.2–12.0)	10.6 (11.6–8.2)^a^	13.2 (14.1–12.3)	11.2 (11.8–10.0)^a^
WC^e^	74.2 (97.0–64.0)	67.6 (74.0–59.0)^a^	71.0 (96.0–60.0)	66.0 (87.0–59.0)^a^	85.0 (104.0–65.0)	66.8 (74.9–56.7)^a^	82.0 (92.5–58.4)	66.8 (77.0–57.0)^a^
BC^e^	95.0 (115.0–75.0)	87.0 (93.0–83.0)^a^	92.8 (112.5–80.0)	88.0 (97.0–81.0)^a^	102.5 (117.5–91.0)	87.5 (92.0–75.0)^a^	106.0 (109.5–85.5)	87.9 (94.0–82.0)^a^
WHR	0.81 (0.89–0.71)	0.79 (0.85–0.69)	0.77 (0.92–0.67)	0.74 (0.90–0.67)^b^	0.82 (0.97–0.68)	0.75 (0.88–0.67)^a^	0.77 (0.88–0.68)	0.73 (0.88–0.70)^b^
WHtR	0.48 (0.63–0.42)	0.44 (0.47–0.37)	0.44 (0.60–0.37)	0.42 (0.52–0.37)^a^	0.54 (0.69–0.41)	0.41 (0.45–0.36)^a^^b^	0.50 (0.61–0.37)	0.43 (0.49–0.39)^a^

*Data are represented as median (upper limit–lower limit);*

c *years*,

d *kg*,

e *cm*,

f *kg/m^2^*,

g *g/dl;*

a *normal vs. anemic*,

b *compared among normal/anemic across ethnic groups; P < 0.05; BMI, body mass index; BW, body weight; WC, waist circumference; BC, buttock circumference; WHR, waist–to-hip ratio; WHtR, waist-to-height ratio*.

### Prevalence of Anemia in Young Malaysian Adults Based on Gender and Ethnicity

The occurrence of anemia among the study groups of subjects is shown in Table 7, considering the hemoglobin concentration of ≥ 13 g per dL in men, ≥ 12 g per dL in women as normal. A total of 50 respondents (20.41%) of 245 were reported to have anemia. Among 113 men and 132 women, a total of 21 (18.6%) men and 29 women (21.96%) were found to be anemic. The gender-wise comparison showed a slightly higher prevalence of anemia in women. While comparing the anemia occurrence across the ethnic groups, it has been found that Indians have the highest prevalence of anemia (26.22%), followed by Malays (21.54%), others (20%), and Chinese (14.5%).

**Table 7:** Distribution of respondents according to their gender, ethnicity, and prevalence of anemia.

	**Total (** ***N*** **=** **245)**		**Malay (** ***N*** **=** **65)**		**Chinese (** ***N*** **=69)**		**Indian (** ***N*** **=** **61)**		**Others (** ***N*** **=** **50)**	
	**Normal**	**Anemic^a^**	**Normal**	**Anemic^a^**	**Normal**	**Anemic^a^**	**Normal**	**Anemic^a^**	**Normal**	**Anemic^a^**
Men (*N* = 113)	92 (81.40)	21 (18.60)	24 (80)	6 (20)	28 (87.5)	4 (12.5)	22 (75.9)	7 (24.1)	18 (81.8)	4 (18.2)
Women (N = 132)	103 (78.04)	29 (21.96)	27 (77.14)	8 (22.86)	31 (83.78)	6 (16.22)	23 (71.8)	9 (28.2)	22 (78.57)	6 (21.43)
Total	195 (79.59)	50 (20.41)	51 (78.46)	14 (21.54)	59 (85.5)	10 (14.5)	45 (73.78)	16 (26.22)	40 (80)	10 (20)

*Data are represented as n (%);*

a *hemoglobin concentration <13 g per dL in men, <12 g per dL in women*.

## Discussion

This is the first study that proposes cut-off values of the major anthropometric indices in predicting the occurrence of anemia in young male and female adults in Malaysia. The study has also evaluated and compared the anthropometric indices and occurrence of anemia among young adults of Malaysia belonging to different ethnicity.

Malaysia homes a large population with anemia with the current prevalence of being 13.8% (Abdullah et al., 2020). A non-invasive simple anthropometric predictor of anemia occurrence will significantly contribute to the early prediction of anemia and thereby aid its prevention. Earlier studies had suggested the essentiality of anthropometry in the prediction of hemoglobin levels under various pathological conditions (Lee et al., 2004; Al-Hashem, 2007; Odagiri et al., 2014; Vuong et al., 2014). This study, using comparative ROC curve with the anthropometric parameters, namely BMI, body weight, WC, WHR, and WHtR as continuous variables and “anemia” as the categorical variable, has found BMI to be the best predictor of the occurrence of anemia among the young adult men (*n* = 113) and women (*n* = 132) of Malaysia (Figure 1). The result attributes to the highest AUC and SP of BMI among all the other anthropometric parameters (Tables 1, 2). The present results are in line with some of the distinguished evidence in this realm. In the Korean population, it was reported that hemoglobin level associated significantly better with BMI and weight as compared with WC and WHtR (Lee and Kim, 2016). In the US population, several studies had reported that low BMI, minimal physical activities, and certain cardiac and renal diseases are among the best predictors of anemia (Frith-Terhune et al., 2000; Zakai et al., 2005; Ausk and Ioannou, 2008; Semba et al., 2009; Neymotin and Sen, 2011). Low BMI was also in the list of the best anemia predictors in the Italian population as described by Cesari et al. (2004). A study in the Chinese population also sinks with our prediction and it stated that obese women had less chances of being anemic than those with normal body weight (Qin et al., 2013). However, there are few studies that supported other anthropometric indices, such as WC (Al-Hashem, 2007), height, and WHR (Lee et al., 2004) over BMI to predict hemoglobin level and occurrence of anemia. In our ROC analysis, among the young adult men in Malaysia, other anthropometric parameters such as the body weight, WC, WHR, and WHtR scored slightly below BMI, all yielding high AUC, SP, and SS in predicting anemia (Figure 1A; Table 1). While, among the young adult women in Malaysia, the values of the anthropometric parameters in predicting anemia had significant differences and can be distinctly arranged in descending order of their efficacy in predicting anemia, which is, BMI > bodyweight> WC > WHtR > WHR (Figure 1B; Table 2). Overall, our findings commensurate with various previous reports (Cesari et al., 2004; Choi et al., 2004; Zakai et al., 2005; Qin et al., 2013) and, thus, it was proposed that BMI is a strong predictor of hemoglobin level and anemia with cut-off values of 20.65 kg/m^2^ and 19.7 kg/m^2^ in young adult men and women of Malaysia, respectively.

In general, anemia is more prevalent in women than in men, mostly because of menstruation, parasitic infestations, physical stress, and inadequate nutrient intake (Piammongkol et al., 2006; Pala and Dundar, 2008; Neymotin and Sen, 2011; Takeda et al., 2011; Abdullah et al., 2017). In this report, the study population comprises young adults who are far from the menopausal age of women of Malaysia, which is reportedly 50 years (Abdullah et al., 2017). It is known that during menstruation, young women normally may lose up to 80 ml of blood per cycle (Magnay et al., 2018). The food intake and dietary iron often fail to compensate for the loss of blood during heavy menstruation resulting in anemia (Mirza et al., 2018). This may explain the higher prevalence of anemia among women than in men in the study (Table 1). Moreover, Malaysia is a multi-ethnic nation, comprising of three main ethnic groups with a sizeable, diverse indigenous population. Based on ethnicity, culture, beliefs, lifestyle, dietary choices vary greatly among the population of Malaysia that deeply influences the physiological status (Gaskell et al., 2008; Hiza et al., 2013; Nohan et al., 2020). Thus, we segregated our data based on the ethnic groups of the young adults of Malaysia (Tables 4–6), and it was seen that the Indians comprised the greatest proportion of the anemic cohort (26.22%) followed by the Malays (21.54%), subjects with indigenous (other) ethnicity (20%), and the Chinese (14.5%) (Table 7). Very few previous reports are available that put forth the variation of anemia occurrence among the different ethnic groups in a multi-ethnic nation (Frith-Terhune et al., 2000; Guralnik et al., 2004; Lau et al., 2015). A study in Singapore, which evaluated anemia in a small number of subjects with chronic kidney disease, had considered ethnicity among its study variables. However, this study did not find any significant differences in anemia among Chinese, Malay, and Indian ethnicities (Lau et al., 2015). Whereas, in the US, a few large-scale studies suggested that the prevalence of anemia due to iron deficiency or low hemoglobin does differ with ethnicity (Frith-Terhune et al., 2000; Guralnik et al., 2004). The study by the National Health and Nutritional Examination Survey (NHANES) showed a significantly higher anemia prevalence among the non-Hispanic black population (Frith-Terhune et al., 2000). Moreover, two recent Malaysian studies also are in congruence with our findings (Yusof et al., 2018; Abdullah et al., 2020). One of the concurrent studies conducted in the Malaysian population aged 35–70 years reported the highest prevalence of anemia among the Malaysian Indians (19.9%), with lower yet concerning prevalence of anemia among the Malays (13.1%) and Chinese (12.0%) (Abdullah et al., 2020). The second study, again based on Malaysian older population aged 60 years or above, showed a high prevalence of anemia in all the three ethnic groups, namely, Indians (42.1%), Malays (36.9%), Chinese (31.1%), and others (36.3%) (Yusof et al., 2018). This study stands unique also for being the first-ever report on the prevalence of anemia and its association with anthropometric indices among young adults in Malaysia.

In summary, this study has proposed specific anthropometric markers with optimal cut-off values to predict anemia among young adults of Malaysia. We envisage that the simple, inexpensive, and intelligible approach of using the anthropometric markers in the prediction of anemia will aid widescale early detection of this crucial health condition and help in the rapid development of its management policies. This analysis reveals that BMI is the most accurate anthropometric marker for the presence of anemia among young adult men and women in Malaysia having the optimal cut-off values of 20.65 kg/m^2^ and 19.7 kg/m^2^, respectively. This study has also shown the pattern of prevalence of anemia based on gender and ethnicity. Future wide-reaching studies in this population of young adults of Malaysia are encouraged that may encompass the association of menstrual parameters and the prevalence of anemia among women. Moreover, studies on the same population, young adults of Malaysia, may also focus on how socioeconomic factors may impact anemia prevalence.

## Data Availability Statement

The raw data supporting the conclusions of this article will be made available by the authors, without undue reservation.

## Ethics Statement

The studies involving human participants were reviewed and approved by Research Management Centre, MAHSA University. The patients/participants provided their written informed consent to participate in this study.

## Author Contributions

SD, IK, and PS have equally contributed to design the study, conceived the study, carried out the experiments, conducted the data analysis, and interpreted the data. SD, IK, PS, and SC have drafted, edited, and reviewed the manuscript. IK has procured the Institutional Ethical Approval for the study. SC has procured the grant for publication. All authors have given their consent for submission.

## Funding

This work was supported by FRGS, Malaysia, FRGS/1/2018/STG03/AIMST/02/1.

## Conflict of Interest

The authors declare that the research was conducted in the absence of any commercial or financial relationships that could be construed as a potential conflict of interest.

## Publisher's Note

All claims expressed in this article are solely those of the authors and do not necessarily represent those of their affiliated organizations, or those of the publisher, the editors and the reviewers. Any product that may be evaluated in this article, or claim that may be made by its manufacturer, is not guaranteed or endorsed by the publisher.
